# Enhancement of the Electroluminescence from Amorphous Er-Doped Al_2_O_3_ Nanolaminate Films by Y_2_O_3_ Cladding Layers Using Atomic Layer Deposition

**DOI:** 10.3390/nano13050849

**Published:** 2023-02-24

**Authors:** Yang Yang, Haiyan Pei, Zejun Ye, Jiaming Sun

**Affiliations:** Tianjin Key Lab for Rare Earth Materials and Applications, School of Materials Science and Engineering, Nankai University, Tianjin 300350, China

**Keywords:** electroluminescence, erbium, Al_2_O_3_, Y_2_O_3_, atomic layer deposition

## Abstract

Amorphous Al_2_O_3_-Y_2_O_3_:Er nanolaminate films are fabricated on silicon by atomic layer deposition, and ~1530 nm electroluminescence (EL) is obtained from the metal-oxide-semiconductor light-emitting devices based on these nanofilms. The introduction of Y_2_O_3_ into Al_2_O_3_ reduces the electric field for Er excitation and the EL performance is significantly enhanced, while the electron injection of devices and the radiative recombination of doped Er^3+^ ions are not impacted. The 0.2 nm Y_2_O_3_ cladding layers for Er^3+^ ions increase the external quantum efficiency from ~3% to 8.7% and the power efficiency is increased by nearly one order of magnitude to 0.12%. The EL is ascribed to the impact excitation of Er^3+^ ions by hot electrons, which stem from Poole-Frenkel conduction mechanism under sufficient voltage within the Al_2_O_3_-Y_2_O_3_ matrix.

## 1. Introduction

Rare earth (RE) ions are generally efficient luminescence centers in various matrices. Nowadays diverse RE-doped insulating materials have been developed for the applications in solid state lasers and phosphors [1,2]. Erbium (Er) ions are one of the most researched luminescence centers due to their near-infrared (NIR) 1.53 μm emission which coincides with the window of optical telecommunication [3,4] Aiming for the realization of Si-integrated optoelectronics, the 1.53 μm electroluminescence (EL) from Er^3+^ ion has been researched extensively in many materials, including SiO_x_, SiN_x_, TiO_2_ and ZnO [5,6,7,8]. However, the efficiencies of the devices based on these aforementioned materials are still far from practical application, due to the limitations in doping tolerance and excitation efficiency. Y_2_O_3_ is one of the attractive doping hosts for RE ions as the substitution of other RE^3+^ ions in Y_2_O_3_ is quite easy without charge compensation and severe lattice distortion. In addition, Y^3+^ ions are not luminescent and Y_2_O_3_ has a large bandgap (5.8 eV) and high stability [9,10]. In our previous study, Al_2_O_3_ has been proved to be a suitable matrix for the excitation of RE^3+^ ion to realize the EL emissions but the doping concentration is still limited [11,12,13]. Therefore, using Y_2_O_3_ as a cladding layer in Er-doped Al_2_O_3_ could utilize the merits of both oxides, the Er-clustering and resultant concentration quenching could be reduced while the optical-active Er^3+^ ions can be excited more effectively [14].

In this work, we fabricate the metal-oxide-semiconductor light-emitting devices (MOSLEDs) based on the amorphous Al_2_O_3_-Y_2_O_3_:Er nanolaminate films, which are deposited using atomic layer deposition (ALD). Due to the unique growth mechanism based on the successive self-limiting gas-surface reactions, ALD realizes the precise control of the thickness of different compositions with excellent homogeneity [15,16]. By alternating deposition sequence of Al_2_O_3_ and Y_2_O_3_, nanolaminate Al_2_O_3_-Y_2_O_3_:Er films with interlayers of different thicknesses are fabricated. Under sufficient forward bias, such devices exhibit ~1530 nm emissions originating from the infra-4*f* transitions of Er^3+^ ions. Inserting of the Y_2_O_3_ cladding layers increases the external quantum efficiency (EQE) from 3% to 8.7% and almost upgrades the power efficiency (PE) by one order of magnitude, while the excitation and recombination of the Er^3+^ ions are not affected. We believe that this work contributes to the development of silicon-based light sources for integrated optoelectronic applications.

## 2. Experimental

The luminescent Al_2_O_3_-Y_2_O_3_:Er nanolaminates were grown on <100>-oriented *n*-type silicon (2–5 Ω·cm) using the thermal ALD system (NanoTech Savannah 100, Cambridge, MA, USA). The growth chamber was first evacuated to a base pressure of 0.3 Torr. Trimethylaluminum [TMA, Al(CH_3_)], Y(THD)_3_ and Er(THD)_3_ (THD = 2,2,6,6-tetramethyl-3,5-heptanedionate) were used as the precursors for Al_2_O_3_, Y_2_O_3_ and Er_2_O_3_, respectively, with ozone acting as the oxidant. During the ALD process, the Al precursor was maintained at room temperature (RT), while Y and Er precursors were maintained at 180 °C and 190 °C, respectively. The precursor delivery lines were heated at 190 °C. N_2_ was used as the carrier and purge gas with a flow rate of 20 sccm. The pulse time for Al and RE precursors are 0.015 s and 2 s, respectively. One growth cycle consists of one precursor pulse, the 5 s N_2_ purge, a 1.8 s ozone pulse, and the 9 s N_2_ purge. Based on the former research, the Er dopant cycles are fixed at 2, which are preferable concerning both the efficient doping and the absence of RE clustering [11,13,17,18]. The substrates were maintained at 350 °C, and the growth rates for the Al_2_O_3_, Y_2_O_3_ and Er_2_O_3_ films are calibrated to 0.79, 0.2 and 0.23 Å/cycle respectively, which agree well with the previous reports [19]. During the deposition, the dopant Er_2_O_3_ atomic layers were sandwiched in two cladding Y_2_O_3_ layers of designed thickness, and then the Al_2_O_3_ interlayers with certain thickness and these Y_2_O_3_-Er_2_O_3_-Y_2_O_3_ composite nanolaminates were deposited repeatedly to achieve the nanolaminates with the deposition sequence of Al_2_O_3_-Y_2_O_3_-Er_2_O_3_-Y_2_O_3_. In order to explore the Al_2_O_3_-Y_2_O_3_:Er nanofilms, firstly for the Al_2_O_3_-Y_2_O_3_:Er nanofilms of different Y_2_O_3_ cladding layers, the thickness of Al_2_O_3_ interlayers was fixed at 3 nm and the two Y_2_O_3_ cladding layers (*x* nm) in each supercycle were changed from 0 to 0.2, 0.5 and 1.0 nm (with their growth cycles varied from 10 to 50), the same repeat numbers of 16 for the supercycles resulted into the total thickness of 48.6, 55.0, 64.6, and 80.6 nm for the Al_2_O_3_-Y_2_O_3_(*x* nm):Er nanolaminates. The calculated nominal doping concentrations of Er are 0.51–0.34 at%. Secondly, for the Al_2_O_3_-Y_2_O_3_:Er nanofilms of different Al_2_O_3_ interlayers, the thickness of Y_2_O_3_ cladding layers were fixed at 0.2 nm and the Al_2_O_3_ interlayers (*y* nm) in each supercycle were changed from 0.5 to 1, 2, 3 and 5 nm. To achieve the Al_2_O_3_-Y_2_O_3_:Er nanofilms of the total thickness of ~65 nm, the repeat numbers of the supercycle were changed from 69 to 45, 27, 19 and 12 for the Al_2_O_3_(*y* nm)-Y_2_O_3_:Er nanolaminates. The calculated nominal doping concentrations of Er are 4.28–0.29 at%. Here the deposition velocities and the growth cycles in recipes, and the densities of oxides (Al_2_O_3_, Y_2_O_3_, Er_2_O_3_) are used to calculate the corresponding dopant amount of Er^3+^ ions. After the deposition, the films were annealed at 800 °C in N_2_ atmosphere for 1 h to enable activation of the dopants. Subsequent device procedures were as previously mentioned [12,13,17,18], resulting in the multilayer-structured MOSLEDs of ZnO:Al/TiO_2_-Al_2_O_3_/Al_2_O_3_-Y_2_O_3_:Er/Si/Al. The top ZnO:Al electrodes were lithographically patterned into 0.5 mm circular dots, while the TiO_2_-Al_2_O_3_ nanolaminates were used to enhance the operation stability of the devices.

The film thickness was measured by an ellipsometer with a 632.8 nm He-Ne laser at an incident angle of 69.8°. The phase and the crystal structure of the films were identified by an X-ray diffractometer (XRD, D/max 2500/pc, Rigaku) using the Cu Kα radiation. To activate EL from the MOSLEDs, appropriate forward bias was applied with the negative voltage connecting to the *n*-Si substrates. EL and Current-Voltage (I–V) characteristics were recorded by a Keithley 2410 SourceMeter. The EL signal was collected by a 0.5 m monochromator and detected by an InGaAs detector connected to a Keithley 2010 multimeter. The absolute EL power from the device surface was measured using a calibrated Newport 1830-C optical power-meter with an 818-IR Sensor. All measurements were performed at RT.

## 3. Results and Discussion

**Figure 1:**
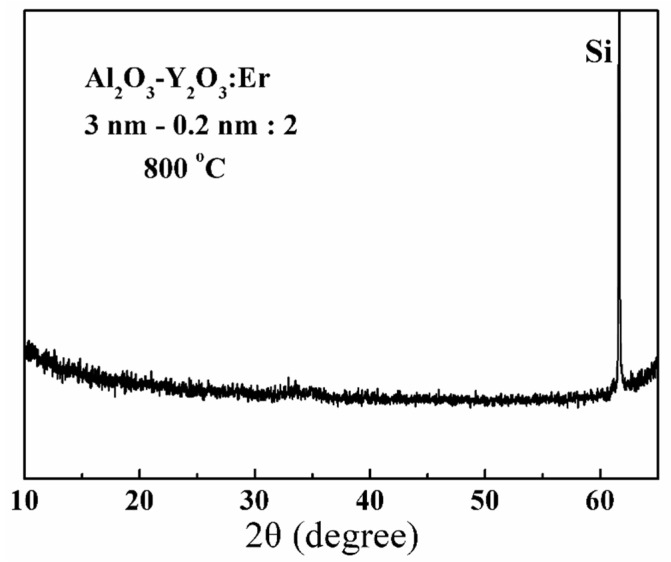
The XRD pattern for the representative Al_2_O_3_-Y_2_O_3_:Er nanolaminate film after annealing at 800 °C.

The XRD patterns of all the Al_2_O_3_-Y_2_O_3_:Er films annealed at 800 °C confirm that the nanolaminates are amorphous, one representative XRD pattern from the nanolaminate using 3 nm Al_2_O_3_ interlayers and 0.2 nm Y_2_O_3_ cladding layers is shown in Figure 1. The Al_2_O_3_ layers are not crystalized at such a relatively low temperature of 800 °C that beneficial for the EL performance from RE-doped Al_2_O_3_ films, while the crystallization of the sub-nanometer Y_2_O_3_ layers is restricted [17,20]. The amorphous nanolaminate films are quite smooth under the observation of scanning electron microscope, with a root-square roughness of only 0.56–0.7 nm scanned by the atomic force microscopy (AFM, Dimension Icon, Bruker) [21].

**Figure 2:**
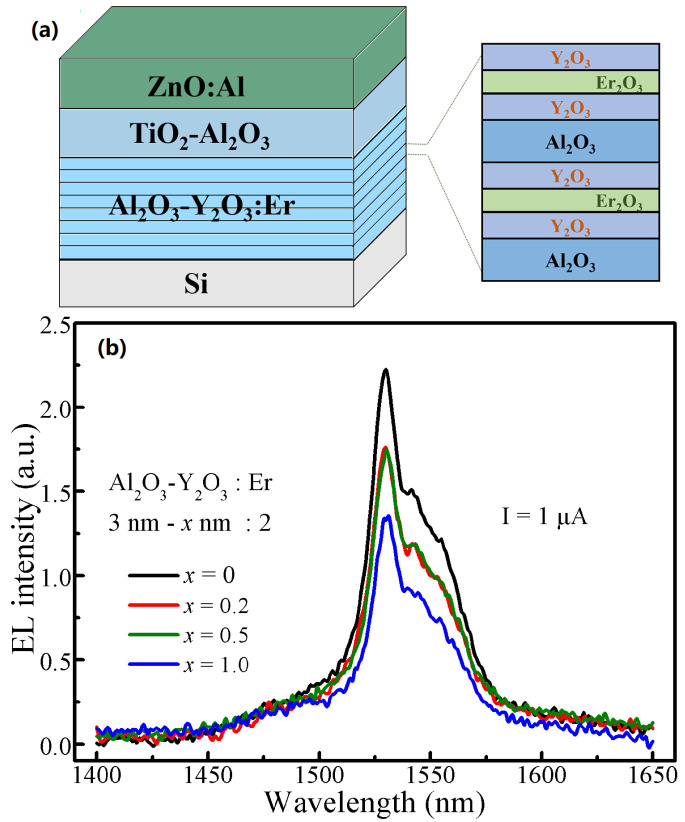
(**a**) The schematic diagram for the Al_2_O_3_-Y_2_O_3_:Er MOSLEDs and the luminescent nanolaminate films. (**b**) The NIR EL spectra for the Al_2_O_3_-Y_2_O_3_:Er MOSLEDs with different Y_2_O_3_ cladding layers (with the thickness of *x* nm) under the injection current of 1 μA.

Figure 2a illustrates the schematic diagram for the MOSLEDs and the structure and deposition sequence of the luminescent nanolaminates. Figure 2b shows the NIR EL spectra of MOSLEDs based on the Al_2_O_3_-Y_2_O_3_:Er films of different Y_2_O_3_ cladding layers (with the thickness of *x* nm). The EL peaks centered at ~1530 nm correspond to the infra-4*f* ^4^I_13/2_→^4^I_15/2_ transitions of the Er^3+^ ions. The presence of other shoulder peaks is ascribed to the splitting levels associated with the Stark effect [22]. These EL peaks are similar in positions and sharps in the Al_2_O_3_-Y_2_O_3_:Er nanofilms with different Y_2_O_3_ cladding layers, thus the incorporation of Y_2_O_3_ cladding layers imposes no apparent effect on the Er^3+^ intra-4*f* transitions. In comparison with the Er-emissions from different matrices, the spectra also confirm that the Al_2_O_3_-Y_2_O_3_:Er films are amorphous due to the absence of companion peaks [20,23].

**Figure 3:**
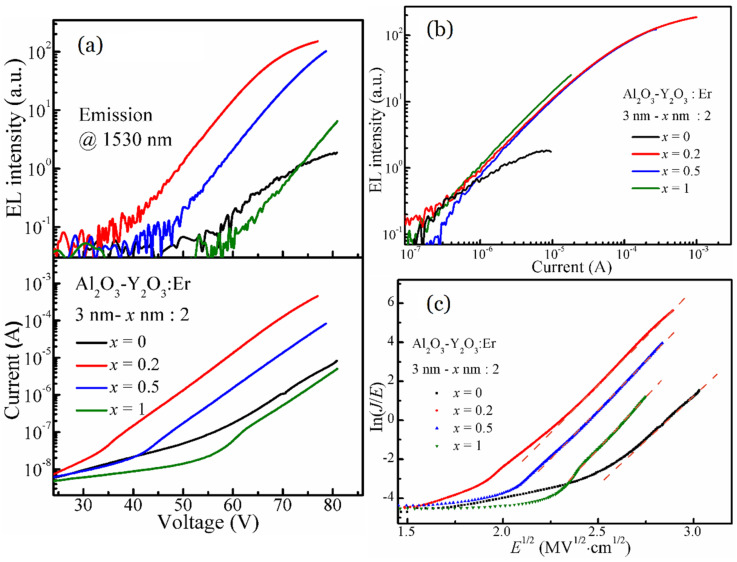
(**a**) The dependence of EL intensities and injection currents on the applied voltage for the Al_2_O_3_-Y_2_O_3_:Er MOSLEDs with different Y_2_O_3_ interlayers (with the thickness of *x* nm), and (**b**) the dependence of EL intensities on the injection currents for these devices. (**c**) The plot of ln(*J*/*E*) versus *E*^1/2^ (P–F plots of the I–V characteristics) for the Al_2_O_3_-Y_2_O_3_:Er MOSLEDs with different Y_2_O_3_ interlayers.

Figure 3a presents the dependence of the 1530 nm EL intensities and the injection currents on the applied voltages for the Al_2_O_3_-Y_2_O_3_:Er MOSLEDs with different Y_2_O_3_ cladding layers (with the thickness of *x* nm). These EL–V and I–V curves are similar with our previous reports on the MOSLEDs based on RE-doped oxides, with the typical characteristic of MOS structures [13,18,24,25]. Beneath the threshold electric field, the defect states contribute to the low background currents. In the working voltage region, the currents increase exponentially until breakdown. The difference on the current injection will be discussed afterwards concerning the conduction mechanism. All the EL intensities also present an exponential relationship with the applied voltages until reaching saturation. The MOSLED with 0.2 nm Y_2_O_3_ cladding layers presents the highest EL intensity, with the lowest threshold voltage and the highest injection current. The devices with thicker Y_2_O_3_ layers underperform in EL intensities and the injection currents are restricted. Despite the uncertainty brought about by the device preparation, Y_2_O_3_ cladding layers with suitable thickness can effectively enhance the current injection and promote the EL emissions from these Al_2_O_3_-Y_2_O_3_:Er MOSLEDs. As previously reported, the incorporation of Y^3+^ ion makes the crystal field around Er^3+^ ions less symmetric and introduces distortion in the crystal field, moreover the Er^3+^ ions are dispersed to suppress the concentration quenching, resulting in the enhanced radiation probability [26]. Therefore, the 0.2 nm Y_2_O_3_ layers act as cladding layers that inhibit the Er-clustering, while the thicker Y_2_O_3_ layers inhibit the electron injection, which is ascribed to the higher dielectric index of Y_2_O_3_ and the disruptive interfaces among Al_2_O_3_ and Y_2_O_3_ interlayers.

Figure 3b shows the dependence of EL intensities on the injection currents for the Al_2_O_3_-Y_2_O_3_:Er MOSLEDs with different Y_2_O_3_ cladding layers. The threshold currents for all the devices are ~0.2 μA, the EL intensities and the injection currents present linear relationship. In comparison, the devices with different Y_2_O_3_ cladding layers exhibit similar EL, which increases more prominently than that based on the Al_2_O_3_:Er film. Y_2_O_3_ also lessens the saturation of EL intensities at higher injection currents. Considering the thick interlayers among RE layers (the Al_2_O_3_ interlayers with the thickness of at least 3 nm), the acceleration distance for hot electrons is sufficient; therefore the enhanced EL should result from more optical-active Er dopants as the Er^3+^ ions disperse into the Y_2_O_3_ layers and the Er-clustering is suppressed.

In our previously reported MOSLEDs based on RE-doped Al_2_O_3_, the RE-related EL is triggered by the direct impact excitation of the RE ions by the hot electrons accelerated under sufficient bias voltages [12,25]. As the I–V characterization are accordingly comparable, it is rational to ascribe the NIR EL from these Al_2_O_3_-Y_2_O_3_:Er MOSLEDs to the same mechanism. Considering the high bandgap of the matrix materials and the barrier for electrons to be injected from the Si substrates into the conduction band of the oxides, the current conduction of these MOSLEDs has been ascribe to the Poole-Frenkel (P-F) mechanism, in which the electrons hop via the defect-related trap states under sufficient electrical field [11,26,27]. In simplicity, the plot of the ln(*J*/*E*) versus *E*^1/2^ presents linear relationship in P-F conduction mechanism, where *J* and *E* are the current density and the electric field, respectively [28,29]. Figure 3c shows the plots of the I–V characteristics derived from Figure 3a, the electrical fields across the luminescent films are roughly calculated in terms of electrostatics [30], and the well-defined linearity is established for all the MOSLEDs in the EL-enabling region. Thus the electron transport through the Al_2_O_3_-Y_2_O_3_:Er nanofilms is governed by the P-F mechanism. These electrons tunnel into the conduction band of oxides and transport by hopping among trap states in the Al_2_O_3_-Y_2_O_3_ nanolaminates under sufficient electric field. Certain parts of the electrons are accelerated therein and become hot electrons that excite the Er^3+^ ions by inelastic impact, the subsequent recombination gives rise to the characteristic EL emissions. Apparently, the Y_2_O_3_ cladding layers decrease the working electric field prominently. As mentioned in the discussion on the I–V characteristics, the Y_2_O_3_ cladding layers increase both the injection currents and EL intensities, we conclude that ultrathin Y_2_O_3_ layers introduce defect sites within Al_2_O_3_, via which electrons transport by the P-F hopping mechanism; therefore the injection currents are enhanced. Since the accelerated electrons collide with the doped Er^3+^ ions and contribute to the NIR EL, adding the aforementioned crystal field distortion and cluster dispersion effects of the Y_2_O_3_ on Er^3+^ ions, the EL performance are greatly enhanced by the Y_2_O_3_ cladding layers in Al_2_O_3_ films. However, the Y_2_O_3_ cladding layers should be thin enough to not impact the carrier transport which could be ascribed to the formation of distinct Al_2_O_3_-Y_2_O_3_ interfaces when using thicker Y_2_O_3_ cladding layers.

**Figure 4:**
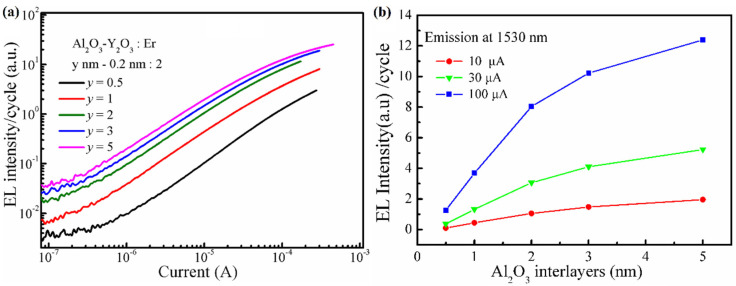
(**a**) The dependence of EL intensities on the injection currents for the Al_2_O_3_-Y_2_O_3_:Er MOSLEDs with different Al_2_O_3_ interlayers (with the thickness of *y* nm), herein the EL intensities are divided by the cycle numbers to manifest the emissions from each Er cycle. (**b**) The integrated EL intensity per cycle as a function of the thickness of the Al_2_O_3_ interlayers under different injection currents.

In evaluation of the thickness of Al_2_O_3_ interlayers on the EL performance, the dependence of the EL intensities from each dopant cycle on the injection currents for the Al_2_O_3_-Y_2_O_3_:Er MOSLEDs with different Al_2_O_3_ interlayers (with the thickness of *y* nm) are shown in Figure 4a, the thickness of Y_2_O_3_ cladding layers is the optimal 0.2 nm. Again, the EL intensities increase almost linearly with the injection currents. The difference on the EL–I–V characteristics among these MOSLEDs with different Al_2_O_3_ layers are small (not shown herein), and the increase in EL intensity with the Al_2_O_3_ thickness could be ascribed to the less concentration quenching of doped Er^3+^ ions together with the longer acceleration distance. When the thickness of Al_2_O_3_ declines, the EL intensity decreases greatly due to the cross-relaxation of Er^3+^ between adjacent dopant layers when the inter-distance (the Al_2_O_3_ thickness) is smaller enough, and the limited acceleration length for the hot electrons to gain energy to excite the Er^3+^ ions [18,21,31,32,33]. Cross-relaxation is a common phenomenon that occurs among the same ions or different ions of similar energy intervals. One ion in the excited state (^4^I_13/2_ in the case of Er^3+^ ion) transfer the energy to another one (in the ground state of ^4^I_15/2_ in this case of Er^3+^ ions), excite the latter to higher energy levels (^4^I_13/2_) while relaxing itself to lower energy levels (^4^I_15/2_) without radiation. The interaction of energy transfer by cross-relaxation could finally disperse the excitation energy through phonons instead of luminescent emissions.

In the RE-doped Al_2_O_3_ MOSLEDs, the Al_2_O_3_ sublayer thickness affects the cross relaxation between excited RE ions, and the acceleration distance for injected electrons. Figure 4b shows the dependence of the integrated 1530 nm EL intensity per Er cycle on the thickness of Al_2_O_3_ interlayers under different injection currents. Under all these injection currents, with the increase in the thickness of Al_2_O_3_ interlayers, the contribution of single Er cycle to the EL intensity firstly increases and then saturates as the Al_2_O_3_ interlayer thickness reaches 3 nm. This is still in consistency with the common characteristic for the luminescent RE^3+^ ions in Al_2_O_3_ matrix that the distance for the presence of non-radiative interaction and adequate electron acceleration is around 3 nm [11,12,21,33].

**Figure 5:**
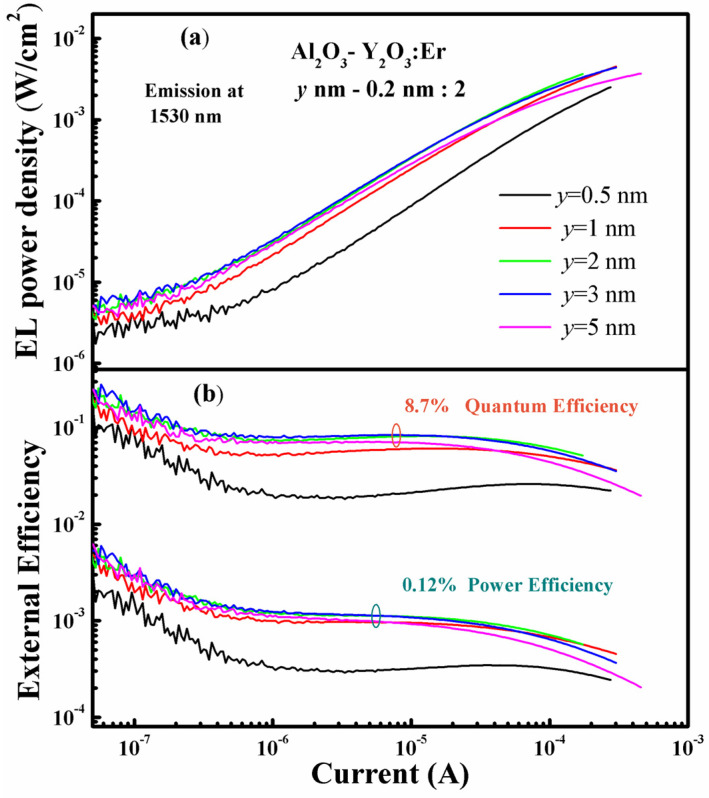
The dependence of (**a**) the EL power densities and (**b**) the external quantum efficiencies (the upper curves) and power efficiencies (the lower curves) on the injection currents for Al_2_O_3_-Y_2_O_3_:Er MOSLEDs using different Al_2_O_3_ interlayers (with the thickness of *y* nm).

Considering the total EL intensity from the MOSLEDs with Al_2_O_3_ interlayers of different thicknesses (marked as *y* nm here) shown in Figure 5a, the device using 3 nm Al_2_O_3_ interlayers presents the optimal emission intensity in the operation range, with the highest power density of 4.6 mW/cm^2^. External efficiency is widely used to evaluate LED performance. Figure 5b shows the EQE and PE of these MOSLEDs based on different Al_2_O_3_-Y_2_O_3_:Er nanolaminate films. These EL efficiencies sustain a broad maximum, and fall down at higher currents. Generally, the EQE of the devices with 2–3 nm Al_2_O_3_ interlayers are the highest. As aforementioned, this phenomenon could be ascribed to the sufficient distance for electron acceleration and suppression of the cross-relaxation among adjacent Er_2_O_3_ dopant layers. The Y_2_O_3_ cladding layers somewhat decrease this critical distance which is beneficial for higher doping concentrations. The optimal device with 3/0.2 nm Al_2_O_3_/Y_2_O_3_ interlayers achieves the maximum EQE of 8.7% and a corresponding PE of 0.12%. These values are comparable to our Yb_2_O_3_:Er MOSLEDs but with lowered working voltages. In comparison, the control Al_2_O_3_:Er MOSLED presents only an EQE of 3% and a PE of 0.014%, much lower than the Al_2_O_3_-Y_2_O_3_:Er MOSLEDs. The Y_2_O_3_ cladding layers with suitable thickness enhance the efficiencies from the MOSLEDs to a great extent. We have found that by using a thicker luminescent layer, the efficiency of the Al_2_O_3_:RE MOSLED might be further increased to higher than 10% [25,34]. These efficiencies are superior to that from Si-based EL devises in literature, thus further optimization of the luminescent Al_2_O_3_-Y_2_O_3_:Er nanolaminates would supply potential light source for the applications in Si-based optoelectronics.

## 4. Conclusions

In summary, significantly enhanced ~1530 nm NIR EL emissions are achieved from the MOSLEDs based on the amorphous Al_2_O_3_:Er nanolaminate films by the insertion of cladding Y_2_O_3_ sub-nanolayers, which are fabricated by ALD on Si substrates. The Y_2_O_3_ cladding layers reduce the threshold electric field for excitation and increase the radiative possibility of doped Er^3+^ ions, resulting in improved EL performance. The Al_2_O_3_-Y_2_O_3_:Er MOSLEDs with 0.2 nm Y_2_O_3_ and 3 nm Al_2_O_3_ interlayers present an EQE of 8.7% and a corresponding PE of 0.12%, which are much higher than that of the counterpart without Y_2_O_3_ cladding layers. The incorporation of Y_2_O_3_ does not change the electron injection mode under sufficient electric field that conforms to P-F mechanism, the resultant energetic electrons trigger the impact-excitation of Er^3+^ ions and subsequent EL emissions. The strategy of Y_2_O_3_-cladding by ALD can be employed to improve the EL performance from LEDs based on RE-doped oxides.

## Data Availability

Data will be made available on request.
